# Characterizing metastatic uveal melanoma patients who develop symptomatic brain metastases

**DOI:** 10.3389/fonc.2022.961517

**Published:** 2022-09-08

**Authors:** Alexander Z. Wei, Matan Uriel, Agata Porcu, Michael P. Manos, Ann C. Mercurio, Michael M. Caplan, Liam Hulse, Rino S. Seedor, Marta Holovatska, Jasmine Francis, Shaheer A. Khan, Diana E. McDonnell, Dmitry Bogomolny, Takami Sato, Brian P. Marr, Rizwan Haq, Marlana Orloff, Alexander Shoushtari, Richard D. Carvajal

**Affiliations:** ^1^ Division of Hematology & Oncology Columbia University Irving Medical Center, New York, NY, United States; ^2^ Department of Medical Oncology Thomas Jefferson University Hospitals, Philadelphia, PA, United States; ^3^ Dana-Farber Cancer Institute, Boston, MA, United States; ^4^ Memorial Sloan Kettering Cancer Center, New York, NY, United States

**Keywords:** uveal melanoma, brain metastases, cutaneous melanoma, mucosal melanoma, acral melanoma, ocular oncology

## Abstract

Metastatic uveal melanoma (mUM) is an advanced ocular malignancy characterized by a hepatotropic pattern of spread. As the incidence of brain metastases (BM) in mUM patients has been thought to be low, routine CNS surveillance has not been recommended. Notably, no formal assessment of BM incidence in mUM has to date been published to support this clinical practice. We aimed to determine the true rate of BM in mUM and to clarify the clinical and genomic risk factors associated with BM patients through a collaborative multicenter, retrospective research effort. Data collected from 1,845 mUM patients in databases across four NCI-designated comprehensive cancer centers from 2006-2021 were retrospectively analyzed to identify patients with BM. Brain imaging in most cases were performed due to onset of neurological symptoms and not for routine surveillance. An analysis of demographics, therapies, gene expression profile, tumor next generation sequencing (NGS) data, time to metastasis (brain or other), and survival in the BM cohort was completed. 116/1,845 (6.3%) mUM patients were identified with BM. The median age at time of UM diagnosis was 54 years old (range: 18-77). The median time to any metastasis was 4.2 years (range: 0-30.8). The most common initial metastatic site was the liver (75.9%). 15/116 (12.9%) BM patients presented with BM at the time of initial metastatic diagnosis. Median survival after a diagnosis of BM was 7.6 months (range: 0.4-73.9). The median number of organs involved at time of BM diagnosis was 3 (range: 1-9). DecisionDX-UM profiling was completed on 13 patients: 10-Class 2, 2-Class 1B, and 1-Class 1A. NGS and cytogenetic data were available for 34 and 21 patients, respectively. BM was identified in 6.3% of mUM cases and was associated with high disease burden and a median survival of under 8 months once diagnosed. Since most patients in this cohort were symptomatic, the incidence of asymptomatic BM remains unknown. These data suggest the use of routine brain imaging in all mUM patients at risk for developing BM for early detection.

## Introduction

Uveal melanoma (UM) is a rare ocular cancer which arises in over 90% of cases from choroidal melanocytes (1). Pigmented cells within the ciliary body (6%) and the iris (4%) can also develop into UM (2). It is more prevalent among males and associated with Caucasians aged 50-70 years old (3, 4). Prevalence around the world is highest in northern European countries with an incidence of 8 per million (5). In the United States, the incidence is approximately 4.6-5 per million with rates being the highest in non-Hispanic whites (3) (6) (4). Risk factors for UM include fair skin, light-colored eyes, northern European ancestry, and sensitivity to sunburns (7) (8).

Approximately 50% of patients with UM will develop metastatic disease within 10 years of primary tumor diagnosis (9) (10). Metastatic UM (mUM) unfortunately confers a poor prognosis of 12-15 months (11) (12) and remains with few treatment options. In January 2022, tebentafusp was approved by the Food and Drug Administration as the first disease-specific treatment for mUM patients however the therapy is restricted to HLA-A*0201 haplotypes (13, 14). The pathogenesis of mUM is associated with two main events: a gain of function mutation in the G_α_ signaling pathway and a secondary alternation – a BAP1, SF3B1 or EIF1AX mutation (“BSE” mutation), which are prognostic for metastatic risk (15). Biallelic inactivation of BAP1 changes regulation in protein de-ubiquitination, cell cycle and apoptosis (16). SF3B1 encodes for the U2 snRNP component of the spliceosome. Mutations in SF3B1 causes abnormal splicing leading to frameshift mutations and mRNA degradation or alternately to activation or change of function mutation (17). Lastly EIF1AX is thought to be a component of the 43s pre-initiation complex responsible to initiate protein translation, but its downstream effects are not completely clear (18). Targeted therapies against downstream effectors of the aforementioned pathways are in various stages of development (19).

In the current era, prognostication and metastatic risk estimation in UM are based upon clinical and genetic factors. The American Joint Committee on Cancer (AJCC) Cancer Staging Manual (8^th^ Edition) includes tumor thickness, location, extraocular extension, and ciliary body involvement. These components were utilized to generate UM staging which increase the 10-year risk of metastasis twofold with each stage. UM thickness in particular has been shown to be an important prognostic factor. Small thickness UM (0-3 mm in thickness) was associated with a 5- and 10-year metastatic risk of 6% and 12%. The 5-/10-year metastatic risk of medium thickness (3.1-8 mm) and large tumors (>8 mm in thickness) was 14%/26% and 35%/49%, respectively (9). Regarding genomic factors, cytogenetic and next generation sequencing-based assays are used to classify metastatic risk. In particular, gene expression profiling (GEP) is a validated method to estimate metastatic risk and categorizes primary UM tumors as Class 1A, Class 1B, and Class 2 disease. Class 1 is divided into two groups: 1A (favorable-risk) and 1B (intermediate-risk) (20). Class 1A GEP tumors carry a main secondary mutation in EIF1AX and have 2 sets of chromosome 8q and partial or total gain of chromosome 6p while class 1B GEP tumors are SF3B1-mutated and have a partial gain of 8q or gain of 6p (21, 22). Class 1A is associated with lower metastatic potential compared to Class 1B with a 5-year metastatic risk of 2% vs 21% (23). Class 2 GEP (high-risk) is characterized by BAP1 loss with cytogenetic aberrations including monosomy 3 or multiple copies of chromosome 8q (24). Class 2 disease is associated with a 72% risk of metastatic disease at 5 years (25).

The pattern of metastasis in UM has been considered to be primarily hematogenous with a marked hepatotropism. Circulating tumor cells (CTC) and tumor DNA (ctDNA) have both been detected in the blood of individuals with mUM (26). However, all patients within the study had radiological signs of metastasis and thus any measurable CTC or ctDNA may simply be a reflection of tumor burden. The most common initial site of metastasis is the liver, with up to 95% of hepatic metastases seen during the course of mUM (10). Factors driving this hepatotropism are not fully clarified, but this phenomenon may in part be explained by known c-MET expression by UM and the prevalence of its associated ligand, hepatocyte growth factor (HGF), in liver viscera (27). Hypothetically, tumor cells can migrate through the hepatic parenchyma, occupy a periportal location, and eventually recruit factors for angiogenesis (28). Additional frequent metastatic sites include the lungs (31%), bones (23%) and soft tissue (17%) (29). As up to 50% of UM patients are at risk for developing metastatic disease, surveillance imaging with abdominal magnetic resonance imaging (MRI) has been recommended for patients with high risk for progression, such as those with BAP1 mutations of Class 2 gene expression profiles (30) (31).

As the incidence of brain metastasis in UM has been considered to be low, routine brain imaging has historically not been recommended. To date, however, the incidence and prevalence of brain metastases (BM) in UM have never been described. All aforementioned prognosticating methodologies include the risk of liver, lung, and bone metastases but do not take BM into account due to limited data availability. BM are believed to be rare, however the clinical characteristics and underlying tumor biology of mUM patients with BM have never been described.

## Materials and methods

### Patients

Deidentified medical data from 2006-2021 were extracted from medical center databases following institutional review board approval. UM patient records were reviewed for the presence of brain metastases (BM) diagnosed at any point in the disease course through MRI scans. Four NCI-designated comprehensive cancer centers within the United States participated in this study in order to create a large, real-world data set for a rare cancer. The secure, institutional databases queried included consecutive patient data at Columbia University, Thomas Jefferson University, Memorial Sloan Kettering Cancer Center, and the Dana-Farber Cancer Institute.

### Study design

Data including patient demographics (gender, age, ethnicity), tumor characteristics (primary site, primary tumor gene expression profile, initial metastatic site, location of metastases, presence of brain metastases, serum lactate dehydrogenase (LDH) level, immunohistochemistry, cytogenetics, metastatic tumor next generation sequencing), treatment history (immunotherapy exposure, lines of therapy), and outcomes (date of primary diagnosis, metastatic diagnosis, and first brain metastasis) were obtained. Brain imaging in almost all cases were performed due to the onset of neurological symptoms and not for routine surveillance. Asymptomatic patients were otherwise detected due to screening brain imaging for clinical trial enrollment. The primary objective of this study was to describe the clinical characteristics of UM patients who develop BM. Secondary objectives included determination of median overall survival (mOS) from primary UM diagnosis, initial metastatic diagnosis, and onset of brain metastasis.

### Statistical analysis

Variables, both continuous and categorical, were summarized with descriptive statistics.

## Results

116 out of 1,845 patients with metastatic uveal melanoma (mUM) developed BM (6.3%) (**Table 1**). Brain imaging was obtained only upon onset of neurological symptoms in most cases of identified brain metastases and not for asymptomatic surveillance. The median age at time of UM diagnosis was 54 years old (range: 18-77). A slight majority of cases occurred in females (54.3%). 89.7% of patients were Caucasian, 4.3% were non-Caucasian, and 6.0% did not report their ethnicities. The majority of primary tumors arose within the choroid (95.2%) followed by iridociliary bodies (2.9%) and the iris (1.9%). Twelve patients did not have initial tumor site location known.

**Table 1:** Clinical characteristics of uveal melanoma patients with brain metastases.

Demographics (n=116)	
Age, median (range)	54 (range: 18-77)
Female (%)	63 (54.3%)
**Primary Tumor Site (n=104)**	
Choroid	99 (95.2%)
Iridociliary bodies	3 (2.9%)
Iris	2 (1.9%)
**Initial Site of Metastases in Patients Who Develop Brain Metastases (n=116)**	
Liver	88 (75.9%)
Bone	21 (18.1%)
Lungs	20 (17.2%)
Brain	15 (12.9%)
Soft tissue	15 (12.9%)
Lymph node	8 (6.9%)
Adrenals	4 (3.4%)
Other organs involved at time of symptomatic brain metastasis, median (range)	3 (range: 0-9)
**Serum Lactate Dehydrogenase at Time of Brain Metastasis (n=86)**	
Normal	30 (34.9%)
>1 ULN (%)	22 (25.6%)
>2 ULN (%)	34 (39.5%)
Elevated LDH (%)	56 (65.1%)
**Treatment (n=114)**	
Lines of therapy prior to brain metastasis, median (range)	3 (range: 0-10)
Treatment with ICI prior to brain metastasis (%)	64 (56.1%)
**Cytogenetics (n=21)**	
Monosomy 3 or 3p deletion (%)	15 (71.4%)
8q amplification (%)	14 (66.67%)
**Gene expression profile by DecisionDX-UM (n=13)**	
Class 1A (%)	1 (7.7%)
Class 1B (%)	2 (15.4%)
Class 2 (%0	10 (76.9%)
**Mutations by Targeted Molecular Panels and Immunohistochemistry**	
BAP1 (n=33)	20 (60.6%)
SF3B1 (n=22)	10 (31.3%)
EIF1AX (n=32)	1 (4.5%)
**Mutations by Next Generation Sequencing (n=34)**	
GNAQ/GNA11	24 (70.1%)
BAP1	5 (14.7%)
PRKCE	4 (11.8%)
MET	4 (11.8%)
CDK2	3 (8.8%)
BCL2	3 (8.8%)
CDKN2A	3 (8.8%)
BRAF	2 (5.9%)
SF3B1	2 (5.9%)
EIF1AX	1 (2.9%)

**Table 1** describes the clinical characteristics of uveal melanoma patients who develop brain metastases at any point during their disease course. LDH, lactate dehydrogenase. ULN, upper limit of normal. ICI, immune checkpoint inhibitor (Anti-PD1, Anti-PD-L1, Anti-CTLA4).

**Figure 1:**
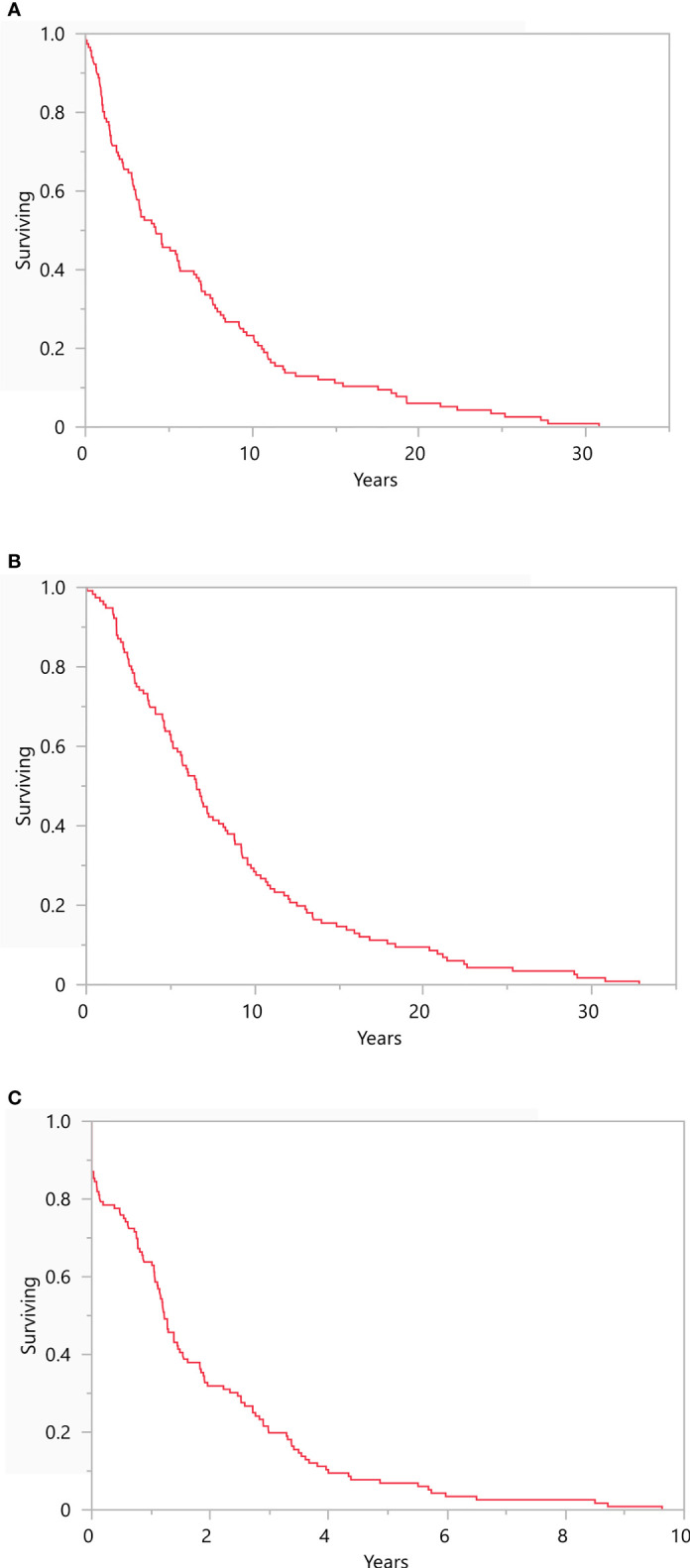
Recurrence Free Survival Kaplan-Meier survival plots representing **(A)** recurrence free survival from primary diagnosis (median 4.2 years, range: 0-30.8), **(B)** development of brain metastasis from primary UM diagnosis (6.5 years, range: 0.04-32.8), and **(C)** development of brain metastasis from initial metastatic UM diagnosis (1.2 years, range: 0-9.6). N = 116.

No patients were diagnosed with BM at the time of diagnosis of the primary tumor, although 1 patient did present with non-BM metastatic disease. The median time to recurrence to mUM was 4.2 years (range: 0-30.8; **Figure 1A**) while the median time to development of BM from primary diagnosis was 6.5 years (range: 0.04-32.8; **Figure 1B**). The median time to development of BM from initial mUM diagnosis was 1.2 years (range: 0-9.6; **Figure 1C**).

**Figure 2:**
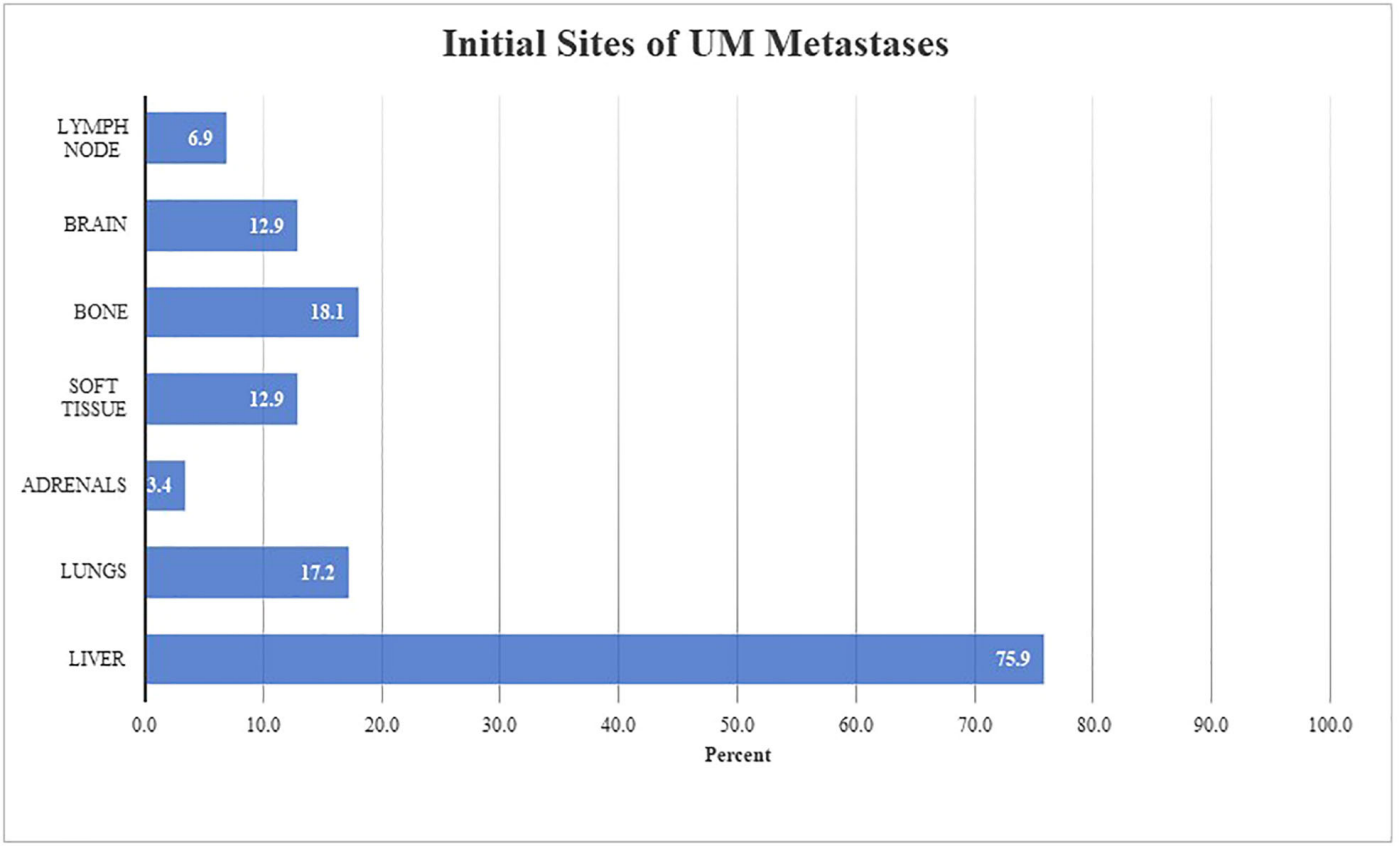
Pattern of initial UM metastasis in patients with brain metastases (n = 116) Figure 2 describes anatomical locations involved at time of initial metastatic diagnosis in our described cohort. Both oligometastatic and synchronous metastatic presentations were observed. The initial site(s) of metastatic disease were liver (n = 88, 75.9%), bone (n = 21, 18.1%), lungs (n = 20, 17.2%), soft tissue (n = 15, 12.9%), brain (n = 15, 12.9%), lymph nodes (n = 8, 6.9%), and adrenals (n = 4, 3.4%).

An analysis was completed to evaluate the pattern of spread in mUM. At time of mUM diagnosis, the most common site of metastasis was the liver (75.9%, n=88) followed by bone (18.1%, n=21), the lungs (17.2%, n=20), soft tissue (12.9%, n=15), and brain (12.9%, n=15) (**Figure 2**). The median number of organ systems involved with tumor was 3 (range: 0-9) at the time of BM. Serum LDH was measured in 86 patients with BM and was elevated in 65.1% of patients (25.6% > 1X ULN and 39.5% >2X ULN).

**Figure 3:**
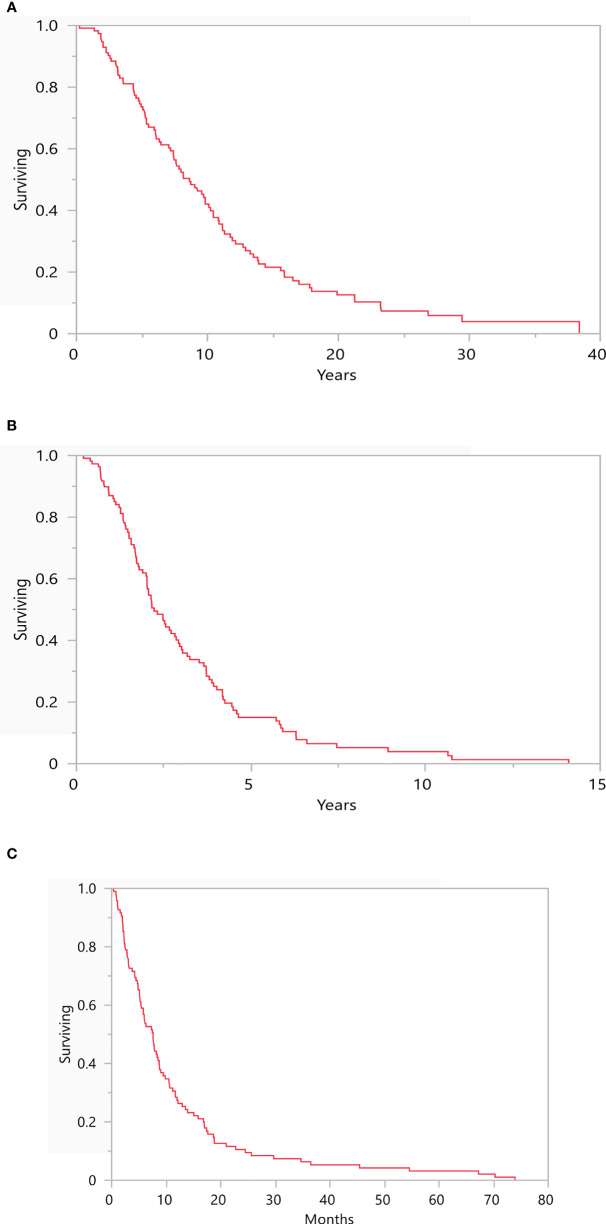
Depicts the median overall survival from **(A)** time of primary diagnosis (8.6 years, range: 0.2-38.4), **(B)** time of initial metastatic disease (2.2 years, range: 0.2-14.1), and **(C)** time of brain metastasis diagnosis (7.6 months, range: 0.4-73.9). N = 116.

At time of data cutoff, 84.8% of patients were deceased (n=95). mOS from primary diagnosis and initial mUM presentation for this cohort were 8.6 years (range: 0.2-38.4) and 2.2 years (range: 0.2-14.1), respectively (**Figures 3A, B**). Following diagnosis of symptomatic BM, mOS was 7.6 months (range: 0.4-73.9; **Figure 3C**).

DecisionDX-UM gene expression profiling (GEP) was completed on the primary tumors of 13 patients who eventually developed BM. 10 patients were found to have Class 2 disease. BAP1, SF3B1, and EIF1AX mutations were detected by immunohistochemistry in 20 out of 33 (60.6%), 10 out of 32 (31.3%), and 1 out of 22 (4.5%) cases respectively. Cytogenetic analysis was available for 21 patients with BM. Monosomy 3 or 3p deletion was detected in 15 samples while chromosome 8q was amplified in 14 cases. Including the former cases, 11 tumor samples had both monosomy 3/3p deletion and 8q amplification. NGS was completed on 34 metastatic tumor samples. 24 of 34 patients (70.6%) had detectable GNAQ/GNA11 mutations. The most notable common non-GNAQ/GNA11 mutations were BAP1 (n=5, 14.7%), MET (n=4, 11.8%), PRKC (n=4, 11.8%), CDK2 (n=3. 8.8%), CDKN2A (n=3, 8.8%), SF3B1 (n=2, 5.9%), and BRAF (n=2, 5.9%) variants.

Previous treatment data from mUM patients with BM were reviewed. Patients received a median of 3 lines of systemic therapy (range: 0-10) prior to developing symptomatic BM. Therapies included both FDA-approved and clinical trial agents. 56.1% of patients received immune checkpoint inhibitors, either as single- or dual agents, prior to BM.

## Discussion

Brain metastases in uveal melanoma are not rare despite prior beliefs. Although no patients in our cohort were diagnosed with BM on initial primary diagnosis, 6.3% of mUM patients ultimately developed BM. Furthermore, nearly 13% of mUM patients who ultimately developed BM presented with BM at time of metastatic diagnosis. The discovery of BM was associated with a poor prognosis of less than 8 months. BM appear to be a late stage of mUM as represented by multi-organ disease involvement at time of diagnosis. Additionally, half of patients had already received multiple lines of systemic therapy, including immune checkpoint inhibitor therapy. As only symptomatic mUM patients were included in this study, the true incidence of BM including asymptomatic patients remains unknown, but is likely higher.

The demographic data from our cohort closely resemble previously reported characteristics (32). The vast majority of UM patients were Caucasian with primary tumors that arose from the choroid. In the metastatic setting, most patients experienced a hepatotropic spread of their cancer. The time course, however, was more varied and represented a more heterogenous disease. The wide range of time between primary and metastatic diagnoses suggest inclusion of patients with deleterious BAP1 mutations associated with an aggressive disease, SF3B1 mutations with late metastatic potential, and other genetic and epigenetic variants which remain unclarified. The mOS for patients with mUM was roughly 2 years, which is longer than purported survival length of 15 months; however, this cohort of patients was enriched for those with later stages of disease.

We attempted to extract genomic data of interest from mUM with BM. Despite multicenter representation, GEP, tumor NGS, and cytogenetics data were only available for 13, 34, and 21 patients, respectively, likely due to their relative novelty and/or accessibility. DecisionDx-UM GEP was available in 2017 and only began widespread use in 2019. Of these 13 patients, the majority of these patients had Class 2 disease, however a bigger sample use will be required to draw any further conclusions about the utility of UM gene expression profiling in predicting BM. Similarly, drawing conclusions from NGS data proved challenging as there remains no accepted, standardized assay for sequencing UM samples. For instance, only 71% of patients had GNAQ/GNA11 mutations detected however it is well known that these mutations are observed in over 90% of UM cases (33), which suggests that these genes may not have been routinely investigated for. Cytogenetic analysis suggested that the majority of patients with BM had either loss of chromosome 3 or chromosome q8 amplification, which are both underlying defects associated with metastatic UM (34, 35). Given the paucity of data in this setting, exploring the genomic and molecular underpinnings of BM development in UM remains an area in need of further investigation.

Our results prompted a comparison to previously described mechanisms and rates of BM development in cutaneous (CM), mucosal (MM), and acral melanomas (AM) (**Table 2**). Multiple studies have reported an incidence of BM in 10% of CM patients, while 40-80% of patients with metastatic CM will develop BM (36, 43). Common risk factors include male gender, age >60, invasive or ulcerated lesions, elevated LDH, and visceral metastasis (44). The development of BM in CM has been strongly associated with the loss of PTEN expression, which leads to increased activation of the PI3K-AKT pathway (45). JAK-STAT signaling and VEGF-A may also play a role in BM development given their effects on blood-brain-barrier permeability and cell growth (46, 47). Additionally, PLEKHA5, which is implicated in brain development, has been implicated as a possible promoter of BM (48). The majority of CM metastases are found in regional lymph nodes; however, common distant metastatic sites include the liver and lungs. This pattern of spread differs significantly from UM where all metastases are typically distant and hepatotropic. Despite this difference, PTEN and other effectors of the PI3K-AKT, JAK-STAT, and VEGF-A signaling pathways should be further investigated as possible underlying mechanisms behind BM in UM. From a clinical perspective, the presence of BM in CM is associated with a poor prognosis of 4-6 months (41), which suggests a more aggressive course when compared to the uveal counterpart described in our report.

**Table 2:** Brain metastases across melanoma subtypes.

	CM	UM	MM	AM
Incidence of BM in metastatic patients	40-60% (36)	6.3%	20-50% (37)	30% (38)
Incidence of BM on initial metastatic diagnosis	30-40% (39)	12.9%	9.2% (40)	Unknown
mOS from BM diagnosis (months)	4-6 (41, 42)	7.6	Unknown	Unknown
Most common initial site of metastasis	Lymph node	Liver	Lymph node	Lymph node

**Table 2** compares the pattern of brain metastases and initial metastases across different melanoma subtypes. CM, cutaneous melanoma; UM, uveal melanoma; MM, mucosal melanoma; AM, acral melanoma.

There is limited literature describing the incidence of BM in MM. Furthermore, there is likely significant heterogeneity in MM depending on the site of anatomical origin (49). A multicenter, retrospective study from France described 21 out of 229 metastatic MM patients (9.2%) had BM at first treatment (40). No data was available describing whether these were oligo-, synchronous, or metasynchronous metastases. A single prospective study of 706 patients describes a metastatic pattern involving a predilection for regional lymphatic (21.5%), pulmonary (21%), and hepatic (18.5%) tissues. No patients with metastatic BM were described. The WHO estimates that the incidence of BM in MM is between 20-50% however this has never been confirmed clinically (37). There is no data regarding molecular markers associated with BM or AM in MM. AM is another rare subset of melanoma with metastatic data equally scarce. One study of 67 metastatic patients (38) described a lymphatic pattern of spread with 94% of patients (n=63) presenting with nodal disease. At time of last follow-up (n=40), 75% of patients (n=30) had developed pulmonary disease followed closely by hepatic (62.5%, n=25) and bone (55%, n=22) involvement. BM was reported in 12 patients (30%).

Given our finding that BM in UM is not uncommon, with 6.3% of mUM patients developing BM and 12.9% of those patients presenting with symptomatic BM at time of metastatic diagnosis, an argument can be made to recommend routine surveillance brain imaging for all patients with UM at the time of initial metastatic diagnosis and at periodic intervals subsequently. Patients with late stages of disease, including 3 or more visceral metastases, a mUM diagnosis over 14 months prior, or treatment failure after 3 systemic lines of therapy should be strongly considered for surveillance brain imaging. Since mUM treatment is currently palliative in intent, an intracranial lesion detected and treated early with regional therapies, including stereotactic radiosurgery (50), could prevent future neurological symptoms and improve quality of life. Future directions of this study include clarifying the underlying molecular mechanisms for the development of BM in UM through tumor whole exome/genome sequencing and, if routine brain imaging occurs, incorporating patients with asymptomatic BM to measure the true incidence of BM in UM.

## Data availability statement

The raw data supporting the conclusions of this article will be made available by the authors, without undue reservation.

## Ethics statement

The studies involving human participants were reviewed and approved by Columbia University Medical Center IRB. Written informed consent for participation was not required for this study in accordance with the national legislation and the institutional requirements.

## Author contributions

AW was responsible for extracting and analyzing data, interpreting results, designing the manuscript, writing the report, updating reference lists, and creating tables/figures. MU was responsible for writing the report and updating reference lists. AP, MM, AM, MC, LH, RS, MH, SK, DM, DB, TS, BM, RH, MO, and AS extracted data and provided valuable feedback for manuscript improvement. RC was responsible for designing the manuscript, writing the report, and providing valuable feedback for manuscript improvement. All authors contributed to the article and approved the submitted version.

## Conflict of interest

Author MO is a consultant for Immunocore, Ideaya, and Delcath and is on the scientific advisory board for Trisalus. Author RC serves as a consultant and/or advisory board member for AstraZeneca, Aura Biosciences, Iconic Therapeutics, Janssen, Merck, Novartis, Rgenix, and Thomson Reuter.

The remaining authors declare that the research was conducted in the absence of any commercial or financial relationships that could be construed as a potential conflict of interest.

## Publisher’s note

All claims expressed in this article are solely those of the authors and do not necessarily represent those of their affiliated organizations, or those of the publisher, the editors and the reviewers. Any product that may be evaluated in this article, or claim that may be made by its manufacturer, is not guaranteed or endorsed by the publisher.
